# Continuing medical education during pandemic waves of COVID-19: Consensus from medical faculties in Asia, Australia and Europe

**DOI:** 10.15694/mep.2021.000064.1

**Published:** 2021-03-08

**Authors:** Carmen Wong, Walter van den Broek, Gillian Doody, Martin Fischer, Michelle Leech, Fabrizio De Ponti, Alexander Gerbes, Hiroshi Nishigori, Young Mee Lee, Maarten Frens, Hideki Kasuya, Franco Bazzoli, Reinhard Hickel, Hong Sik Lee, J.P.T.M van Leeuwen, Christina Mitchell, Kenji Kadomatsu, John Atherton, Francis Chan

**Affiliations:** 1 The Chinese University of Hong Kong; 2 Erasmus University Medical Center; 3 University of Nottingham; 4 Ludwig Maximilian University of Munich; 5 Monash University; 6 University of Bologna; 7 Nagoya University; 8 Korea University

**Keywords:** COVID-19, pandemic, infection control, teaching, medical faculties, consensus, guidance

## Abstract

This article was migrated. The article was marked as recommended.

Medical faculties have the responsibility to train tomorrow’s doctors and in a crisis face the challenge of delivering students into the workforce promptly and safely. Worldwide, medical faculties have faced unprecedented disruptions from viral outbreaks and pandemics including SARS, Ebola, H1N1 and COVID-19 which bring unique challenges. Currently there is worldwide disruption to medical faculties and medical education due to COVID-19. Despite close links with clinical medicine and the known risks of pandemics, many medical faculties have been caught off guard without pandemic planning in place, to deal with an exponential rise in infections and deaths, overwhelmed health services and widespread community risk of transmission. Assessing transmission risk of COVID-19 in teaching, clinical and community attachments and continuing medical education is paramount as medical faculties face subsequent pandemics waves. Consensus statements based on best available evidence and international expertise from medical faculties in Asia, Australia and Europe were developed to help guide the protection of staff and students, priorities on teaching activities and further educational development. Infection prevention, infection control, contact tracing and medical surveillance are detailed to minimise transmission and to enhance safety. Recommendations on teaching activities planning can enhance responsiveness of medical faculties to tackle subsequent waves of COVID-19 infection. A global approach and dialogue are encouraged.

## Introduction

The initial alert of the novel coronavirus in Wuhan, China on 31 December 2019 and subsequent community transmission ( Huang *et al.,* 2020) was initially under the radar of many medical schools across the world. As epidemics erupted in Iran, Italy and transmitted across Europe, United States and globally, medical faculties had to respond rapidly to regional and national containment and mitigation efforts as well as ensuring the safety of medical staff, medical students and patients. The timing of COVID-19 pandemic had varying impacts on teaching and assessment arrangements in different countries and medical faculties. The COVID-19 pandemic is estimated to persist for many more months into 2021 with researchers suggesting episodic quarantine and lockdown as a possible strategy to reduce transmission until a vaccination is available and widespread ( Ferguson *et al.,* 2020).

Medical schools are now experiencing the impact of this on their curriculum planning and infection control measures in the longer term as they anticipate further waves of COVID-19 infections in the near future. In Western countries, approximately 10% of health care professionals have been infected with COVID-19 ( Remuzzi and Remuzzi, 2020; Ng *et al.,* 2020) health care facilities and intensive care units were overwhelmed, resulting in exponential rise in infection and deaths with a large national and regional diversity ( Xie *et al.,* 2020). In addition, clinical staff had to deal with inadequate or insufficient personal protective equipment (PPE), lack of ventilators and make difficult treatment decisions ( Truog *et al.,* 2020) resulting in ongoing physical risks and psychological toil with many cases of physical and psychological exhaustion as health care workers became infected, quarantined or unwell ( Chen *et al.,* 2020; Cai *et al.,* 2020). This has and will continue to have a direct impact on capacity of medical faculties to provide teaching and assessments as well as the provision of experienced examiners. Many medical schools, noting previous infection risks to students during SARS ( Patil and Yan, 2003; Clark, 2003) have suspended face to face clinical and community teaching as the pandemic evolved. Some schools have opted to forgo or postpone the final clinical examinations and graduating students early ( Amante and Balmer, 2003; General Medical Council, 2020) and discuss whether to continue online learning into the next academic year.

Current knowledge about COVID-19 includes substantial pre-symptomatic and asymptomatic transmission ( Bai *et al.,* 2020; Zha ng *et al*., 2020; Zhou *et al.,* 2020) with an average 3 infections (range of 1.4 -6.5) from a single infection ( Liu *et al.,* 2020), although transmission rates can vary depending on the national and regional situation. COVID-19 has been shown to affect all ages but serious illness or death mostly occur in the elderly and those with comorbidities such heart disease, lung disease and diabetes ( Emami *et al.,* 2020; Guo *et al.,* 2020). Main transmission routes appear to be by droplets transmission to close contacts or contact with contaminated surfaces, although in some circumstances, there is evidence of aerosol transmission. Emerginig information about infection risk and transmission of COVID-19 allows medical faculties to can make measured responses in optimizing staff, communications and infection control to better provide medical education particularly in clinical settings. As further pandemic waves of COVID -19 looms, a clear and thoughtful policy regarding infection prevention and control, prioritisation of class resumption and planning for further waves of infections will be required. Through the Global Alliance of Medical Excellence (GAME) is an international collaboration of medical faculties, consensus guidance from medical faculties in Asia, Australia and Europe in continuing medical education cover three main topics:

1. How to protect staff and medical students during the COVID-19 viral pandemic?
2. What teaching and assessment activities should be prioritised?
3. How can medical faculties evolve to enhance medical education for subsequent pandemic waves?

## Method

A Pubmed/MEDLINE search was performed using ‘severe acute respiratory distress syndrome’ ‘SARS’ ‘COVID-19’ ‘pandemic’ ‘pandemic planning’ ‘medical education’ ‘medical schools’ ‘medical students’ as MeSH terms. Statements from the international Medical Associations (e.g. American Medical Association, British Medical Association etc.), Medical College Associations e.g. Association of American Medical Colleges etc.) and international medical bodies such as the World Health Organization and Centers’ for Disease Prevention and Control were prioritized. In addition, opinion and perspectives articles of medical journals were also discussed to stimulate consensus.

Guidance was grouped according to WHO pandemic phase descriptions: Phase 4 - 6, post peak and containment, delay and mitigation ( World Health Organisation, 2020a). Phase 4 is sustained human to human transmission, Phase 5 and 6 are peak of the pandemic where there is widespread transmission.

Containment phase aims to prevent disease transmission by early detection, isolation, medical surveillance with contact tracing and screening e.g. in Hong Kong, Australia. Delay phase aims to slow the spread and lower and delay the peak of infected cases. Measures include social distancing, closure of schools and cancellation of mass gatherings e.g. in United Kingdom, The Netherlands and United States. The mitigation phase occurs once infection is widespread and optimises medical health care personnel and facilities to optimise care e.g. in China, Iran, Italy ( World Health Organisation, 2020a).

All member institutes were invited to contribute and respond to statements and consensus was reached when agreement was reached in ≥ 75 % of all member institutions. Member institutions were asked to state the degree of agreement (strongly agree, agree with minor reservations, agree with major reservations, disagree with major reservations, strongly disagree). Statements, tables and figures which reached ≥ 75 % agreement (strongly agree, agree) were retained and a second round of revisions were made similarly by consensus. Details of member institutions who responded are detailed in Appendix 1.

## Recommendations

### Part 1: How to protect faculty staff and medical students during COVID-19 pandemic

#### General Comments

1. A rapid response team for pandemic response should be set up to review regular updates and coordinate the pandemic response ( Centers for Disease Control and Prevention, 2020a). Wherever possible, this should include a multidisciplinary team of public health specialists, medical educators/ academic year coordinators, communications experts and clinicians active in service. Staff with experience in pandemic planning or in response to previous epidemic or pandemics (e.g. SARS, Ebola etc.) can be helpful.
2. Student exchanges to affected countries should be terminated immediately and medical schools should assist students in returning to their home or study country as soon as possible. Future student exchanges may be risk assessed as the pandemic evolves but all student exchanges should be suspended if there are any cases of global infection ( Medical Schools Council, 2020a). Medical schools may need to seek guidance from government for evacuation assistance.
3. Medical schools should act in accordance with national guidelines. If no guidance is given, all curricular clinical and community attachments should be suspended at the first detection of infection regionally ( Medical Schools Council, 2020a) and remain suspended until there are no signs of local or community acquired infection for the recommended 14 day period ( Larsen *et al.,* 2020). Final year students and those volunteering in community electives and activities may be supported by medical schools following careful evaluation of risks, infection control and hospital and community needs.
4. A voluntary self-reporting surveillance system should be set up for students and staff. Details of the surveillance includes travel history and plans, contact with COVID-19 patients or high-risk individuals (e.g. returning residents from affected countries), daily signs and symptoms e.g. fever, cough etc., place of residence. The surveillance system allows easy identification, monitoring and risk assessment for work/study at home and return to work/schools/ examinations ( Centre for Disease Control and Prevention, 2020a). State or national policy may influence decision of medical schools to adopt voluntary surveillance.
5. All staff and students should receive communication on infection and prevention control for COVID-19. This should include updated advice about potential sources of contamination, use of facemasks, handwashing or use of alcohol gel >70%, social distancing, self-isolation, help seeking and availability and criteria for testing ( Centre for Disease Control and Prevention, 2020a).
6. Medical faculties should liaise with hospital/clinic partners and facilitate feedback processes, evaluation and incident reporting from staff and students about infection control measures.
7. Staff and students may also be vulnerable to mental ill health e.g. anxiety from the threat of COVID-19, ill health or death of a loved one, self-isolation etc. ( Al-Rabiaah, 2020; Chen *et al.,* 2020). Adequate counselling support should be available e.g. dedicated mental health team or counsellor and capacity for student support can be enhanced with extending the roles of mentors, academic advisors and teachers.

#### Faculty staff and facilities

1. Delineate essential work staff and roles and initiate work at home policies. Work at home policies will be influenced by state and national guidelines e.g. during lockdown. For essential workers ensure social distancing for those who return to office e.g. stagger start and finish times to avoid crowding at public transport and distancing of office space. Offer technical support and guidance for those working at home ( Centers for Disease Control and Prevention, 2020a).
2. COVID-19 (SARS -CoV-2) can persist on paper for 2 days and hard surfaces e.g. glass, stainless steel for 4-7 days ( Chin *et al.,* 2020) and can be inactivated by bleach and common disinfectants ( World Health Organisation, 2020b). Routine disinfection in medical schools may be guided by state or national regulations. In the absence of guidance and if resources are available, it is recommended that common spaces and facilities and areas of high use e.g. handrails, lift buttons and door handles should be rroutinely cleaned and disinfected ( U.S. Department of Health & Human Services, 2020).
3. Staff should triage their symptoms daily and only enter office areas if they are symptom free and use appropriate respiratory protective equipment such as the use of facial masks. The use of FFP1 or cloth masks should be used if available ( World Health Organisation, 2020b; Department of Health, 2020; Feng *et al.,* 2020). FFP2 /3 masks should be used in higher risk clinical contact areas, provided there is ample sample and hospital use has been prioritised. The wearing of facial masks will be influenced by state and national guidelines.
4. Regular updates regarding step up and step-down pandemic work arrangements should be communicated as far ahead as possible ( Centers for Disease Control and Prevention, 2020a).
5. Medical schools should be aware of staff working in separate hospitals or units taking care of infected patients or patients with high suspicion of infection and minimise contact with other faculty staff and students to reduce the risk of infection to medical students.

#### Medical students

1. Effective communication is vital. Utilise effective modes of communication and connection with students e.g. regular liaison with class representatives and regular communications should be made to manage expectations as the pandemic evolves. Timely communication about surveillance, infection control and prevention can lessen anxiety. A named contact person or unit should be made available for student queries and assistance ( Centers for Disease Control and Prevention, 2020a).
2. Low risk teaching areas e.g. off-site non-clinical facilities such as simulation teaching areas can be identified in advance as reserve facilities for use when there is limited community transmission and satisfactory containment measures. Additional infection control and prevention measures such disinfection, social distancing, small groups may be used to further reduce infection risk ( Centers for Disease Control and Prevention, 2020a).
3. Careful consideration regarding which examinations are deemed essential is critical and the option of and feasibility for online assessment and should be encouraged e.g. remote testing using open book format to test critical thinking. If essential examinations are conducted on site during low risk periods of the pandemic strict measures including surveillance of examiners, supporting staff and students, triage, social distancing and regular disinfection are required (See Table 1). Resources for onsite examination will become limited in the mitigation phase. In this phase staff resources may be low and there is increased infection risk in patients and simulated patients and asymptomatic COVID-19 carriers. In this phase may need to be delayed or alternative/ online arrangements should be sought.
4. Medical schools are responsible for students’ safety in campus, community or clinic/ hospital-based activities and should maintain close liaison with teaching hospitals and clinic. Medical schools have an ethical responsibility to provide adequate guidance for students who volunteer e.g. competencies, safety, infection control ( Harvey, 2020; Medical Schools Council, 2020b; Whelan *et al.,*2020a; Whelan *et al.,*2020b). Health or community partners should provide indemnification for volunteer placements and early employment.

**Table 1.** Infection control and prevention for on-site essential examinations

Period	Procedure	Details
Preparation	Medical surveillance	Mandatory completion of online health declaration of travel history, contact history and symptoms by supporting staff, examiners and students
	Examination criteria and logistics	**Written examinations** Revisit format of examinations e.g. written versus multiple choice to reduce handling of test paper Ensure physical distancing of 1.5m between students. Consider multiple venues for conducting examination **Clinical examinations** Simulated patients should be used instead of real patients and deemed infection free (e.g. self- declaration of symptoms and travel/ contact history +/- testing) Reduce number and duration of test stations if possible Ensure social distancing of 1.5m or more between students and staff and examiners. Reduce length of time and number of students in waiting areas
	Ventilation	Examination rooms, waiting areas and test stations should be well ventilated
	Liaison and communication	Inform students of examination procedures in advance Arrange online briefing session to respond to student queries
	Crowd control	Stagger student registration times Utilise multiple register areas or venues
Examination	Coordination	Support staff and examiners should be clear about their roles, infection controls and tasks. A central coordinator or coordinating team is required to ensure procedures and processes.
	Personal protection	All staff, students and examiners should wear respiratory facial protection. FFP1 or cloth facial mask should be used. FFP2/3 can be used if in ample supply ^a^. This measure could be influenced by state and national guidelines
	Triage	Use infrared temperature probe for screening and identification of staff, students and examiners with a temperature > 37.5 Celsius. A second tympanic temperature probe can provide a more accurate reading for confirmation. Those who are confirmed with temperature > 37.5 Celsius would not be allowed to continue with the examination and advised to seek medical assistance.
	Hand hygiene	Hand sanitisers with alcohol content >70% ^b^ should be readily available in examination rooms and waiting areas. If hand sanitisers are not available, easy access to handwashing facilities are necessary.
	Environmental disinfection	Supporting staff should disinfect high traffic areas and surfaces with 1: 99 diluted bleach ^c^. Provision of individual alcohol wipes to students can enhance disinfection of the student’s immediate vicinity.
	Personal belongings	Students should place personal belongings in designated guarded area for self-collection or on a trolley if transferred to a different venue
Post examination	Environmental disinfection	All areas and surfaces including test devices e.g. table computers/ laptops should be disinfected with 1:100 dilution of 5% sodium hypochlorite or alcohol wipes > 70% alcohol ^d^

^a^ World Health Organisation, 2020b; Department of Health, 2020; Feng *et al*., 2020

^b^ World Health Organisation, 2014

^c^ Centers for Disease Control and Prevention, 2002

^d^ World Health Organisation, 2014; Centers for Disease Control and Prevention, 2020b

As the pandemic evolves, there is a need to step-up and step-down infection control measures. Table 2 details recommendations based on the current updates about COVID-19.

**Table 2.** Infection control: Pandemic phases and key actions for medical faculties for COVID-19

Phase 4 Sustained human to human transmission		
No regional cases	Observation	Set up rapid response team and review updates
First regional case	Containment	Follow national guidelines (if any) Consider suspend all international students exchanges and local/ regional community and clinical attachments Consider setting up an online medical surveillance for staff and students Commence online learning
**Phase 5 & 6 Widespread human infection** Regular communication and updates to staff and students. Mental health support for staff and students Mandatory respiratory protection (facial mask), physical distancing and regular office disinfection		
Minimal or limited disruption to community activity	Containment	Consider continuation of essential examinations Routine +/- flexible home office working
Limited community activity	Delay	Rota of essential office staff, flexible hours, work from home. Postpone examinations or seek alternatives.
Lockdown and essential activities only	Mitigation	Work from home. Key worker in office only. Postpone examinations or seek alternatives.
**POST PEAK PERIOD Level of pandemic drops below pandemic level. Possibility of recurrent events** Continue regular communication and mental health support for staff and students Continue mandatory respiratory protection (facial mask), physical distancing and regular office disinfection		
No cases or sporadic/ cluster of cases Minimal or limited disruption to community activity	Containment	Liaise with hospital to assess readiness for teaching Consider restarting essential teaching and examinations Routine +/- flexible home office working
Sustained human transmission Limited community activity	Delay	Rota of essential office staff, flexible hours, work from home
**POST PANDEMIC Disease activity at seasonal levels** Regular office disinfection.		
No cases or sporadic/ cluster of cases Minimal or limited disruption to community activity	Containment	Resume face to face teaching Regular communication and activation of medical surveillance when required

### Part 2: Prioritising teaching and assessment activities during COVID-19

1. When face to face teaching in community and clinical attachments is suspended, course coordinators should review and revise teaching activities and assessments in line with learning outcomes.
2. Medical faculties should support rapid staff development in online teaching and learning for teachers and students. Support may be technological e.g. internet access, provision of notebooks, webcams, microphones, technical assistance for online learning and video conferencing platforms e.g. use of platform features, setting up video communications as well as educational support e.g. use of enhanced program features, exploring different teaching models. The capacity for individual support and guidance require need sufficient resources. Training and use of department support staff can also help problem solve common issues.
3. Sufficient time should be given for online curriculum planning and team meetings, which can enhance a collaborative approach and efficient use of strengths and skills within the team.
4. Online modalities can be effective for lectures, case based, problem based and team-based learning ( Abrahamson, 2006; Augestad and Lindsetmo, 2009; Barrows, 1996; Joyce *et al.,* 2017; Lim *et al.,* 2006; Lim *et al.,* 2009; Wong *et al.,* 2005) Interactive sessions can be enhanced by using small groups and multimodal teaching including patient and virtual simulation (see Table 3).
5. As COVID-19 pandemic evolves and with the likelihood of recurrent community transmission, the strategy should prioritise key tasks which require face to face teaching during the period of no or low transmission with capacity in delivery (see Table 4.)

**Table 3.** Enhancing online teaching

Effective	Strategies to enhance student engagement
Recorded lectures Live lectures Tutorials Flipped classroom Case based scenarios Problem based learning Team based learning Roleplays Communication Skills Video vignettes Simulated patients via video	Use of chat feature can enable students to ask questions - for large groups, a facilitator (i.e. staff or peer student) can help monitor the questions and responses. Polls can help teachers and students gauge understanding. Use short small group sessions with online video can enhance interaction Consistent grouping of students can help group dynamics and cohesion: to this end, organising breakout rooms and the role of digital moderator (ie student representatives or junior staff) can be very useful to help peer-to-peer interaction during online lteaching sessions A consistent tutor can further gauge student’s progress and offer feedback. Virtual clinics using constructed cases and multimodal tasks can help students to contextualise cases seen e.g. photograph of clinic, patient or clinical signs, excerpts from clinical notes, roleplay, data interpretation etc. Many universities have simulated patient programmes and activities. History taking skills can be developed in novice learners through the inclusion of simulated patient in tutorials. Plan content with the view to share recorded sessions of different teachers and cases between different groups to enhance learning. For roleplays in communication skills, assign alternating roles in advance and email corresponding scripts for preparation. Use of material on the internet e.g. videos on physical examination, patient stories and clinical case based teaching can augment the learning experience. Student presentations and demonstrations can help assess understanding in clinical procedures and assessment.
**Limited effectiveness** Clinical procedures Physical examinations Clinical assessment Community activities Service learning	

**Table 4.** Priorities and adaptations in medical education during COVID-19

Priority	Suggested adaptations
**High Priority** Final year clinical examinations Final year written examinations Final year clinical teaching on wards/clinics Final year high fidelity simulation of complex cases e.g. intensive care, acute care Clinical teaching and examination in other years	Change written examination format to viva or computerised adaptive testing which can be done online or opportunistic delivery using multiple venues. Opportunistic clinical examination delivery with strict infection control measures Change clinics to virtual clinics (See Table. 3) with real case scenarios of that clinic session and incorporate clinical tasks e.g. history taking, clinical assessment and counselling. Describe and demonstrate procedures and examinations
**Normal priority**	Active adaptation to online learning, resume face to face when able (subject to national policy, community activity restriction and infection risk). Schedule courses most amenable to online learning e.g. anatomy, biochemistry, radiology etc. first over clinical courses in peak and post peak pandemic periodContinue simulated patient teaching and preclinical teaching and use of cadavers in small groups with infection control and social distancing when amenable
**Low priority** Asynchronous courses with established online format Community/ service learning e.g. attachment with community non-governmental organisations, home visits.	Use programmatic approach and self-directed learning for established asynchronous online courses Postpone community visits. When visits can resume, distribute experiences between student groups and use presentations and sharing to enhance learning and reflection. Build a library of online interviews with non-governmental organisations and patient interviews

### Part 3: Enhancing medical education

1. The mass adoption of online teaching and learning brings opportunities for collaborative efforts in content development e.g. clinical case and clinical signs/ photos library, patient narratives. This is particularly useful for countries in which medical schools share curriculum or qualifying examinations e.g. United State, Canada, Germany. Further discussion is necessary on minimal competency frameworks and the feasibility and adaptation of national clinical exams in further waves of COVID-19 infection. Meanwhile, there is potential for international collaboration to expand the cultural and diversity and development of global resources for medical education.
2. Pandemic preparedness is particularly pertinent for final year students as they prepare to work during COVID-19 pandemic. Evidence of pandemic preparedness teaching in medical schools have largely been theoretical, with teaching and learning during COVID-19 pandemic logistically and technically challenging ( Li *et al.,* 2020). There is a role for medical schools to liaise with local hospital and primary care networks to establish key areas and tasks in which medical schools can assist to train students. This can include opportunities for clinical case studies, medical ethics as well as infection control and PPE training.
3. During COVID-19, suggested roles for medical students include assisting with childcare for health professionals, assisting the elderly to participating in low risk clinical tasks ( Lee, 2020; Miller *et al.,* 2020), whilst there are evident needs where additional help is required e.g. in nursing homes ( Orrechio-Egrestiz, 2020). Much less is known about the medical students’ attitudes and willingness to volunteer. Further research and discussion are needed to explore opportunities for learning and how medical schools can facilitate learning roles in placements or alternatives e.g. telemedicine, protect students in at-risk areas and enhance social responsibility efforts and roles in the community.

## Conclusion

In this evolving COVID-19 pandemic, medical training must continue to ensure an uninterrupted competent workforce and the recommendations set out this consensus guidance can help medical schools prepare and facilitate teaching and assessment with guidance on infection control for staff and students, enhancing online teaching and prioritising teaching activities as well the need for research and development to enhance medical education to withstand future pandemic waves of COVID-19.

## Take Home Messages

• As COVID-19 pandemic evolves, medical faculties must continue medical education to ensure an uninterrupted competent workforce.
• This consensus guidance developed from medical faculties in Asia, Australia and Europe of current best evidence and expertise can help medical schools prepare and facilitate teaching and assessment in subsequent pandemic waves of COVID-19.
• Guidance covers infection control measures for faculty staff and students, recommendations in teaching modalities and planning as well as suggestions for further faculty and educational development.
• A global approach and dialogue in tackling subsequent waves of COVID-19 is encouraged.

## Notes On Contributors

**Prof. Carmen Wong** is Assistant Dean of Education at the Chinese University of Hong Kong in Hong Kong SAR China. ORCID ID: https://orcid.org/0000-0003-2164-3673

**Prof. Walter van den Broek** is Director of Medical Education at Erasmus Medical Centre in Rotterdam, The Netherlands.

**Prof. Gillian Doody** is Dean of Medical Education at University of Nottingham in the United Kingdom.

**Prof. Martin Fischer** is Associate Dean for Students at Ludwig Maximilian University of Munich in Germany.

**Prof. Michelle Leech** is Deputy Dean of the Medicine Nursing and Health Sciences Faculty at Monash University in Australia.

**Prof. Fabrizio De Ponti** is Professor at the Department of Medical and Surgical Sciences, University of Bologna in Italy.

**Prof. Alexander Gerbes** is Professor at the Faculty of Medicine at the Ludwig Maximilian, University of Munich in Germany.

**Prof. Hiroshi Nishigori** is Associate Professor at the Center for medical education at Nagoya University in Japan.

**Prof. Young Mee** **Lee** is Professor at the Department of Medical Education in Korea University in Korea.

**Prof. Maarten Frens** is Professor at Erasmus University Medical Center in the Netherlands.

**Prof. Hideki Kasuya** is Associate Dean of International Affairs at Nagoya University in Japan.

**Prof. Franco Bazzoli** is Professor of Gastroenterology at the University of Bologna in Italy.

**Prof. Reinhard Hickel** is Professor and Dean at Ludwig Maximilian University of Munich in Germany.

**Prof. Hong Sik Lee** is Professor at the Department of Medical Education in Korea University in Korea.

**Prof. J.P.T.M van Leeuwen** is Dean and vice-chairman Executive Board Erasmus Medical Centre in the Netherlands.

**Prof. Christina Mitchell** is Dean of Faculty of Medicine Nursing and Healthcare sciences at Monash University in Australia.

**Prof. Kenji Kadomatsu** is Professor at Nagoya University Graduate School of Medicine in Japan.

**Prof. John Atherton** is Pro Vice Chancellor and Dean of Faculty of Medicine and Health Sciences at the University of Nottingham in United Kingdom.

**Prof. Francis Chan** is Dean of Faculty of Medicine at the Chinese University of Hong Kong in Hong Kong SAR China.

## Figures and Tables

**Appendix 1:** Medical institutions: student intake and initial faculty pandemic response

Institution, Country	Student intake per year (duration of study)	Faculty Pandemic Response (from January 2020)
The Chinese University of Hong Kong, Hong Kong SAR, China	230-240 students 6 years	Fully virtual teaching by 1 February 2020. Staff work at home arrangements. Essential staff arrangements in office. All clinical examinations successfully conducted March - April 2020 (Medical years 4-6) during second wave of infection. Staff resume normal office hours May 4 ^th^ 2020.
LMU Munich, Bavaria, Germany	870-880 students (preclinical yrs. 1 + 2), 470-500 (clinical yrs. 3-6), 6 years	Fully virtualized semester started April 20 ^th^, selected exams on a voluntary basis on Campus allowed when hygiene rules can be secured, stay in home office whenever possible.
Korea University College of Medicine, Seoul, South Korea	106 students 6 years	Faculty members and staffs of university hospitals and medical college have worked on-site, not at home for all periods of COVID-19. However the entire curriculum has transformed to online format since Feb. 25th. Clinical rotations were suspended for three weeks and recommenced from March 16 ^th^. The face to face cadaveric dissection recommenced on April 21. Anatomy and Physiology mid-term examinations were conducted face to face on April 16 ^th^, and 20th, respectively. All deferred examination and simulation sessions are planned to conduct by face to face around early July. At the entire university level, KU recommended online lecture for the semester, but small group sessions less than 30 students was allowed form May 4 ^th^, only if students agree with their attendance.
Monash School of Medicine, Melbourne, Australia	500 students 5 years	Campus based activity fully online and faculty deliver teaching from home. Hospital based staff work from in situ clinical school facilities. Senior students continue in hospital placements.
Erasmus MC, Rotterdam, The Netherlands	410 students 6 years	Lockdown Medical education fully remote teaching for bachelor students. Last year of master has restarted their clerkships
University of Bologna, Bologna, Italy	350 students for each of the 6 years (Italian stream)Approx.. 60 students for each year (English stream)	Lockdown Medical education: fully remote teaching started in March (lessons; oral and written exams; graduation]. Staff allowed to work from home whenever possible; apart from clinical activities, staff must justify circumstances requiring their presence in the University premises.
Nagoya University, Japan	680-190 students 6 years	Online remote classes onlyBoth undergraduate and graduate students are prohibited from coming to the university. Graduate students engaged in ongoing experiments and research activities are permitted.
University of Nottingham, United Kingdom	485 4 years 5 years 6 years	Lockdown since 23 ^rd^ March. University premises closed, no access unless special permission granted. All staff working from home. Immediate online teaching for early years and postgraduate students. Online learning provided for clinical years from 6 ^th^ April. Early graduation of final year students (exams Feb), virtual resits. Medical Faculty Staff co-opted into NHS service.

## References

[ref1] Abrahamson S. D. Canzian S. and Brunet F. (2006) Using simulation for training and to change protocol during the outbreak of severe acute respiratory syndrome. Critical Care (London, England). 10(1),R3. 10.1186/cc3916 16356209 PMC1550819

[ref2] Al-Rabiaah A. Temsah M.-H. Al-Eyadhy A. A. Hasan G. M. (2020) Middle East Respiratory Syndrome-Corona Virus (MERS-CoV) associated stress among medical students at a university teaching hospital in Saudi Arabia. Journal of Infection and Public Health. 13(5), pp.687–691. 10.1016/j.jiph.2020.01.005 32001194 PMC7102651

[ref3] Amante A. and Balmer C. (2020) Italy Rushes New Doctors Into Service as Coronavirus Deaths Rise Above 2,500, Reuters. Available at: >https://www.reuters.com/article/%20us-health-coronavirus-italy/italy-rushes-to-promote-new-doctorsto-relieve-coronavirus-crisis-idUSKBN214245( Accessed: 1 August 2020).

[ref4] Augestad K. M. and Lindsetmo R. O. (2009) Overcoming Distance: Video-Conferencing as a Clinical and Educational Tool Among Surgeons. World Journal of Surgery. 33(7), pp.1356–1365. 10.1007/s00268-009-0036-0 19384459 PMC2691934

[ref5] Bai Y. Yao L. Wei T. Tian F. (2020) Presumed Asymptomatic Carrier Transmission of COVID-19. JAMA. 323(14), p.1406. 10.1001/jama.2020.2565 32083643 PMC7042844

[ref6] Barrows H. S. (1996) Problem-based learning in medicine and beyond: A brief overview. New Directions for Teaching and Learning. 1996(68), pp.3–12. 10.1002/tl.37219966804

[ref7] Cai H. Tu B. Ma J. Chen L. (2020) Psychological Impact and Coping Strategies of Frontline Medical Staff in Hunan Between January and March 2020 During the Outbreak of Coronavirus Disease 2019 (COVID‑19) in Hubei, China. Medical Science Monitor: International Medical Journal of Experimental and Clinical Research. 26, e924171. 10.12659/MSM.924171 32291383 PMC7177038

[ref8] Centers for Disease Control and Prevention . (2002) Guidelines for hand hygiene in healthcare settings. Available at: >https://www.cdc.gov/infectioncontrol/guidelines/hand-hygiene/(Accessed: 1 August 2020).

[ref9] Centers for Disease Control and Prevention . (2020a) Interim Guidance for Administrators of U.S. Institutions of Higher Education. Plan, Prepare, and Respond to Coronavirus Disease 2019 (COVID-19). Available at: https://www.cdc.gov/coronavirus/2019-ncov/downloads/guidance-administrators-college-higher-education.pdf( Accessed: 1 August 2020).

[ref10] Centers for Disease Control and Prevention.(2020b) Interim Recommendations for U.S. Households with Suspected or Confirmed Coronavirus Disease 2019 (COVID-19). Cleaning and Disinfection for Households. Available at: https://www.cdc.gov/coronavirus/2019-ncov/community/organizations/cleaning-disinfection.html( Accessed: 1 August 2020).

[ref11] Chen Q. Liang M. Li Y. Gui J. (2020) Mental health care for medical staff in China during the COVID-19 outbreak. The Lancet Psychiatry. 7(4),e15–e16. 10.1016/S2215-0366(20)30078-X 32085839 PMC7129426

[ref12] Chin A. W. H. Chu J. T. S. Perera M. R. A. Hui K. P. Y. (2020) Stability of SARS-CoV-2 in different environmental conditions. The Lancet Microbe. 1(1), e10. 10.1016/S2666-5247(20)30003-3 32835322 PMC7214863

[ref13] Clark J. (2003) Fear of SARS thwarts medical education in Toronto. BMJ (Clinical Research Ed.). 326(7393), p.784. 10.1136/bmj.326.7393.784/c PMC1169342 12689971

[ref14] Department of Health. (2020) Guidelines on prevention of coronavirus disease 2019 (COVID-19) for the general public.Department of Health, Hong Kong S.A.R. Available at: https://www.chp.gov.hk/files/pdf/nid_guideline_general_public_en.pdf( Accessed: 1 August 2020).

[ref15] Emami A. Javanmardi F. Pirbonyeh N. and Akbari A. (2020) Prevalence of Underlying Diseases in Hospitalized Patients with COVID-19: a Systematic Review and Meta-Analysis. Archives of Academic Emergency Medicine. 8(1), e35. 32232218 PMC7096724

[ref16] Feng S. Shen C. Xia N. Song W. (2020) Rational use of face masks in the COVID-19 pandemic. The Lancet Respiratory Medicine. 8(5), pp.434–436. 10.1016/S2213-2600(20)30134-X 32203710 PMC7118603

[ref17] Ferguson N. M. Laydon D. Nedjati-Gilani G. Imai N. (2020) Impact of non-pharmaceutical interventions (NPIs) to reduce COVID19 mortality and healthcare demand’, Imperial College COVID-19 Response Team, London, March 16 2020. Available at: >https://www.imperial.ac.uk/media/imperial-college/medicine/sph/ide/gida-fellowships/Imperial-College-COVID19-NPI-modelling-16-03-2020.pdf( Accessed: 1 August 2020).

[ref18] General Medical Council . (2020) Joint statement: early provisional registration for final year medical students. Available at: https://www.gmc-uk.org/news/news-archive/early-provisional-registration-for-final-year-medical-students( Accessed: 1 August 2020).

[ref19] Guo T. Fan Y. Chen M. Wu X. (2020) Cardiovascular Implications of Fatal Outcomes of Patients With Coronavirus Disease 2019 (COVID-19). JAMA Cardiology. 5(7), pp.811–818. 10.1001/jamacardio.2020.1017 32219356 PMC7101506

[ref20] Harvey A. (2020) Covid-19: medical students should not work outside their competency, says BMA. BMJ (Clinical Research Ed.). 368, m1197. 10.1136/bmj.m1197 32209588

[ref21] Huang C. Wang Y. Li X. Ren L. (2020) Clinical features of patients infected with 2019 novel coronavirus in Wuhan, China. The Lancet. 395(10223), pp.497–506. 10.1016/S0140-6736(20)30183-5 PMC7159299 31986264

[ref22] Joyce M. F. Berg S. and Bittner E. A. (2017) Practical strategies for increasing efficiency and effectiveness in critical care education. World Journal of Critical Care Medicine. 6(1), pp.1–12. 10.5492/wjccm.v6.i1.1 28224102 PMC5295164

[ref23] Larsen D. Dinero R. E. Asiago-Reddy E. Green H. (2020) A review of infectious disease surveillance to inform public health action against the novel coronavirus SARS-CoV-2. https://doi.org/10.31235/osf.io/uwdr6

[ref24] Lee Y. J. (2020) Medical Students Around the US Are Offering to Babysit for Hospital Workers on the Frontlines of the Coronavirus Pandemic. New York, NY: Business Insider. Available at: https://www.businessinsider.com/medical-students-babysit-healthcare-workers-covid-19-coronavirus-2020-3( Accessed: 1 August 2020).

[ref25] Li Li Lin M. Wang X. Bao P. and Li Y. (2020) Preparing and responding to 2019 novel coronavirus with simulation and technology-enhanced learning for healthcare professionals: challenges and opportunities in China. BMJ Simulation & Technology Enhanced Learning. 6(4), pp.196–198. 10.1136/bmjstel-2020-000609 PMC7410111 32832099

[ref26] Lim E. C. H. Oh V. M. S. Koh D.-R. and Seet R. C. S. (2009) The challenges of “continuing medical education” in a pandemic era. Annals of the Academy of Medicine, Singapore. 38(8), pp.724–726. 19736579

[ref27] Lim E. C. H. Ong B. K. C. and Seet R. C. S. (2006) Using videotaped vignettes to teach medical students to perform the neurologic examination. Journal of General Internal Medicine. 21(1), p.101. 10.1111/j.1525-1497.2005.00271_2.x PMC1484627 16423134

[ref28] Liu Y. Gayle A. A. Wilder-Smith A. and Rocklöv J. (2020) The reproductive number of COVID-19 is higher compared to SARS coronavirus. Journal of Travel Medicine. 27(2). 10.1093/jtm/taaa021 PMC7074654 32052846

[ref29] Medical Schools Council (2020a) Advice from Medical Schools Council to UK Medical Schools on actions surrounding Covid 19. Available at: https://www.medschools.ac.uk/media/2620/msc-covid-19-advice-for-uk-medical-schools.pdf( Accessed: 1 August 2020).

[ref30] Medical Schools Council (2020b) Statement of expectation. Medical student volunteers in the NHS. Available at https://www.medschools.ac.uk/media/2622/statement-of-expectation-medical-student-volunteers-in-the-nhs.pdf( Accessed: 1 August 2020).

[ref31] Miller D. G. Pierson L. and Doernberg S. (2020) The Role of Medical Students During the COVID-19 Pandemic. Annals of Internal Medicine. 173(2), pp.145–146. 10.7326/M20-1281 32259194 PMC7151405

[ref32] Ng K. Poon B. H. Kiat Puar T. H. Li Shan Quah J. (2020) COVID-19 and the Risk to Health Care Workers: A Case Report. Annals of Internal Medicine. 172(11), pp.766–767. 10.7326/L20-0175 32176257 PMC7081171

[ref33] World Health Organisation . (2014) Infection prevention and control of epidemic- and pandemic-prone acute respiratory infections in health care: WHO guidelines. Available at: https://www.who.int/publications/i/item/infection-prevention-and-control-of-epidemic-and-pandemic-prone-acute-respiratory-infections-in-health-care(Accessed: 1 August 2020). 24983124

[ref34] Orrechio-Egrestiz H. (2020) As many as half of Europe’s COVID-19 deaths were people in long term facilities, Business Insider. Available at: https://www.businessinsider.com/half-europes-covid-19-deaths-in-long-term-care-facilities-2020-4( Accessed: 1 August 2020).

[ref35] Patil N. G. Chan Y. and Yan H. (2003) SARS and its effect on medical education in Hong Kong. Medical Education. 37(12), pp.1127–1128. 10.1046/j.1365-2923.2003.01723.x 14984121 PMC7168501

[ref36] Remuzzi A. and Remuzzi G. (2020) COVID-19 and Italy: what next? The Lancet. 395(10231), pp.1225–1228. 10.1016/S0140-6736(20)30627-9 PMC7102589 32178769

[ref37] Teixeira P. and Shin J. C. (eds.) (2020) Encyclopedia of international higher education systems and institutions. Dordrecht: Springer.

[ref38] Truog R. D. Mitchell C. and Daley G. Q. (2020) The Toughest Triage - Allocating Ventilators in a Pandemic. The New England Journal of Medicine. 382(21), pp.1973–1975. 10.1056/NEJMp2005689 32202721

[ref39] U.S. Department of Health & Human Services . (2020) Cleaning and Disinfecting Your Facility. Available at: https://www.cdc.gov/coronavirus/2019-ncov/community/disinfecting-building-facility.html( Accessed: 1 August 2020).

[ref40] Whelan A. Prescott J. Young G. Catanese V. M. (2020) Guidance on Medical Students’ Participation in Direct Patient Contact Activities, Association of American Medical Colleges. Available at: https://www.aamc.org/system/files/2020-08/meded-August-14-Guidance-on-Medical-Students-on-Clinical-Rotations.pdf( Accessed: 1 August 2020).

[ref41] Wong M. L. Koh D. Phua K. H. and Lee H. P. (2005) Teaching community, occupational and family medicine at the National University of Singapore: past, present and future. Annals of the Academy of Medicine, Singapore. 34(6):102C–107C. 16010387

[ref42] World Health Organisation . (2020a) Operational planning guidelines to support country preparedness and response. Available at: https://www.who.int/docs/default-source/coronaviruse/covid-19-sprp-unct-guidelines.pdf( Accessed: 1 August 2020).

[ref43] World Health Organisation . (2020b) WHO Coronavirus disease (COVID-19) advice for the public: when and how to use masks. Available at: https://www.who.int/emergencies/diseases/novel-coronavirus-2019/advice-for-public/when-and-how-to-use-masks( Accessed: 1 August 2020).

[ref44] World Health Organisation . (2014) Annex G, Use of disinfectants: alcohol and bleach. Infection Prevention and Control of Epidemic- and Pandemic-Prone Acute Respiratory Infections in Health Care. Geneva: World Health Organization. Available at: https://www.ncbi.nlm.nih.gov/books/NBK214356/( Accessed: 1 August 2020). 24983124

[ref45] Xie J. Tong Z. Guan X. Du B. (2020) Critical care crisis and some recommendations during the COVID-19 epidemic in China. Intensive Care Medicine. 46(5), pp.837–840. 10.1007/s00134-020-05979-7 32123994 PMC7080165

[ref46] Zhang J. Litvinova M. Wang W. Wang Y. (2020) Evolving epidemiology and transmission dynamics of coronavirus disease 2019 outside Hubei province, China: a descriptive and modelling study. The Lancet Infectious Diseases. 20(7), pp.793–802. 10.1016/S1473-3099(20)30230-9 32247326 PMC7269887

[ref47] Zhao S. Gao D. Zhuang Z. Chong M. K. C. (2020) Estimating the Serial Interval of the Novel Coronavirus Disease (COVID-19): A Statistical Analysis Using the Public Data in Hong Kong From January 16 to February 15, 2020. Frontiers in Physics. 8. 10.3389/fphy.2020.00347

