# Supplementary material for: Continuing medical education during pandemic waves of COVID-19: Consensus from medical faculties in Asia, Australia and Europe
Source: MedEdPublish (2016). 2021 Mar 8;10:64. [Version 1] doi: 10.15694/mep.2021.000064.1 (PMC10939633; doi:10.15694/mep.2021.000064.1)
Supplement: Peer review history

# Reviewer report: Reviewer response for version 1

**DOI:** 10.21956/mep.18917.r27039

**Published:** 2021-03-21

**Authors:** Samar Aboulsoud

**Affiliations:** 1 CAIRO UNIVERSITY, SCHOOL OF MEDICINE

This review has been migrated. The reviewer awarded 4 stars out of 5 I would like to thank the prestigious group of authors for sharing this well-thought review. I found the recommendations very useful with many tips that are relevant to the majority of undergraduate medical programs across the globe.I have only one comment and a suggestion.1. The title of the article (CME) doesn’t reflect the content (UGE) and this might be misleading for the readers and researchers. 2. There are clearly two themes in the paper; the protective approach for the faculty and students (infection control) and the educational enhancement techniques. Although they seem to be two sides of the same coin, however and given the importance of the topic, the authors might like to consider expanding on the two themes and splitting them into two separate papers. Thank you again for sharing this valuable information

Reviewer Expertise:

NA

No decision status is available

# Reviewer report: Reviewer response for version 1

**DOI:** 10.21956/mep.18917.r27040

**Published:** 2021-03-14

**Authors:** Balakrishnan Nair

**Affiliations:** 1 Centre for Medical Professional Development and University of Newcastle

This review has been migrated. The reviewer awarded 4 stars out of 5 This is a well thought out and researched article on how to continue undergraduate medical education in the face of the current pandemic. The authors are very senior academics and the lead many medical education programs. The article can guide others to manage medical education programs when faced with such emergencies. This gave us an opportunity to reflect on what we have done and what else we could have done during this crisis . The title can be misleading . “Continuing Medial Education “ usually means CME and implies continuing professional development for practising physicians. May be “Maintaining Undergraduate Medical Education ---- “ or similar would have been more accurate

Reviewer Expertise:

NA

No decision status is available
